# Ulceroglandular form of tularemia after squirrel bite: a case report

**DOI:** 10.1186/s13256-022-03510-8

**Published:** 2022-08-17

**Authors:** Hannah Sophia Borgschulte, Daniela Jacob, Jörg Zeeh, Holger C. Scholz, Klaus Heuner

**Affiliations:** 1 Department of Internal Medicine 2, St. Katharinen-Hospital, Kapellenstr. 1-5, 50226 Frechen, Germany; 2 Division of Highly Pathogenic Microorganisms (ZBS 2), Centre for Biological Threats and Special Pathogens, Robert Koch Institute, Seestr. 10, 13353 Berlin, Germany

**Keywords:** Tularemia, Zoonosis, *Francisella tularensis* subspecies *holarctica*, Eurasian red squirrel, Case report

## Abstract

**Background:**

The diagnosis of tularemia is not often considered in Germany as the disease is still rare in this country. Nonetheless, *Francisella tularensis*, the causative agent of tularemia, can infect numerous animal species and should, therefore, not be neglected as a dangerous pathogen. Tularemia can lead to massively swollen lymph nodes and might even be fatal without antibiotic treatment. To our knowledge, the case described here is the first report of the disease caused by a squirrel bite in Germany.

**Case presentation:**

A 59-year-old German woman with a past medical history of hypothyroidism and cutaneous lupus erythematosus presented at the emergency room at St. Katharinen Hospital with ongoing symptoms and a swollen right elbow persisting despite antibiotic therapy with cefuroxime for 7 days after she had been bitten (right hand) by a wild squirrel (Eurasian red squirrel). After another 7 days of therapy with piperacillin/tazobactam, laboratory analysis using real-time polymerase chain reaction (PCR) confirmed the suspected diagnosis of tularemia on day 14. After starting the recommended antibiotic treatment with ciprofloxacin, the patient recovered rapidly.

**Conclusion:**

This is the first report of a case of tularemia caused by a squirrel bite in Germany. A naturally infected squirrel has recently been reported in Switzerland for the first time. The number of human cases of tularemia has been increasing over the last years and, therefore, tularemia should be taken into consideration as a diagnosis, especially in a patient bitten by an animal who also presents with headache, increasing pain, lymphadenitis, and fever, as well as impaired wound healing. The pathogen can easily be identified by a specific real-time PCR assay of wound swabs and/or by antibody detection, for example by enzyme-linked immunosorbent assay (ELISA), if the incident dates back longer than 2 weeks.

## Background

Here, we present the case of a 59-year-old German woman who was admitted to our hospital after a squirrel bite with an ulcerative lesion at digit IV of the right hand and a lymphadenitis at the right elbow combined with persistent fever, myalgia, and headache. The diagnosis of tularemia was based on the results of a specific real-time polymerase chain reaction (PCR) assay that showed positivity for *Francisella tularensis*, and on culture of the material on agar plates showing growth typical of *Francisella*.

Tularemia, also called “rabbit fever,” is a rare, but potentially severe zoonosis caused by *F. tularensis*. The disease has been described in more than 250 animal species, including mammals, birds, amphibians, fish, and invertebrates, all of which potentially transmit the bacterium to humans. Transmission of *F. tularensis* to humans may also occur through mosquito or tick bites and through the consumption of contaminated water or food [1–5]. Human-to-human transmission has not yet been reported [6]. Four *F. tularensis* subspecies (*tularensis*, *holarctica*, *mediasiatica* and *novicida*) care known to cause tularemia, but only two, *F. tularensis* ssp. *tularensis* and *F. tularensis* ssp. *holarctica* (*Fth*), are of clinical relevance. To our knowledge, however, the only *F. tularensis* subspecies identified in Germany so far is *Fth* [7]*.* In endemic regions, especially, hunters and forest workers are at an increased risk for tularemia. Because of the low prevalence of the disease in Germany [8], physicians might be unaware or underestimate the disease, which leads to delayed adequate diagnoses and specific treatment [9–12].

Only rarely can the sources of human infections be verified. For example, during a recent outbreak that was linked to freshly pressed grape must, a wood mouse was identified as a potential source of contamination [9, 11, 13]. To our knowledge, the case described here is the first report in Germany of a human infection caused by a squirrel bite occurring, although a naturally infected squirrel has also been reported from Switzerland recently [14].

## Case presentation

A 59-year-old German woman with a past medical history of hypothyroidism and cutaneous lupus erythematosus presented at the hospital´s emergency room 7 days after having been bitten by a wild Eurasian red squirrel (*Sciurus vulgaris*).

The patient reported having noticed a squirrel lying motionless on the ground when she took a walk. As soon as she tried to examine the animal, it started biting her, biting digit IV of the right hand and digit II of the left hand. After the wounds had been cleaned and dressed, she was started the same day on antibiotic therapy consisting of cefuroxime 500 mg orally twice a day. Tetanus vaccination status was also checked. On day 6 after the bite, the patient presented at the hospital’s emergency room showing the following symptoms: general malaise, aching head and body, fever, chill, and a swollen right elbow. She did not report having suffered from nausea, vomiting, or sensitivity to light or noise.

The clinical examination showed a minor bite on digit IV of the right hand, located near the middle phalanx, slightly reddened, yet without clear inflammatory signs. There was no pain to pressure, no movement restriction, nor any sensory deficit. There were also visible signs of lymphadenitis at the ulnar side of the right elbow. The peripheral sensitivity as well as movement skills and blood flow were intact. At the index finger of the left hand, another minor bite without any local or proximal signs of inflammation was recorded. The physical examination did not reveal anything unusual. The vital parameters were stable, with the exception of a negligible sinus tachycardia. No signs of meningism were found.

Laboratory tests showed an increase in the C-reactive protein (CRP) up to 28.6 mg/L (reference < 5 mg/L), but no leukocytosis. The test for procalcitonin (indicating bacterial inflammation) was negative. Sampled cerebrospinal fluid and urine were also negative in standard laboratory tests. X-rays of the thorax and of digit IV of the right hand and a native computed tomography (CT) brain scan did not show any pathological outcomes. Real-time PCR assays for severe acute respiratory syndrome Coronavirus 2 (SARS-CoV-2), respiratory syncytial virus, and influenza A and B were negative. Starting on day 7, the medical treatment also comprised combination therapy with intravenous antibiotics (ampicillin/sulbactam, 2 g/1 g) 3 times a day. However, the patient’s condition did not improve and fever rose up to 39.2 °C and persisted until day 12. Blood culture remained negative, even at 14 days after inoculation. CRP increased up to 185 mg/L on day 12. As a consequence, treatment was intensified by commencing combination antibiotic therapy with piperacillin/tazobactam (4 g/0.5 g) on day 11. Followin initiation of therapy with piperacillin/tazobactam, the CRP fell to 74 mg/L (day 14) and there was a mild persisting leukocytosis (12.14 × 10^3^).

**Fig. 1:**
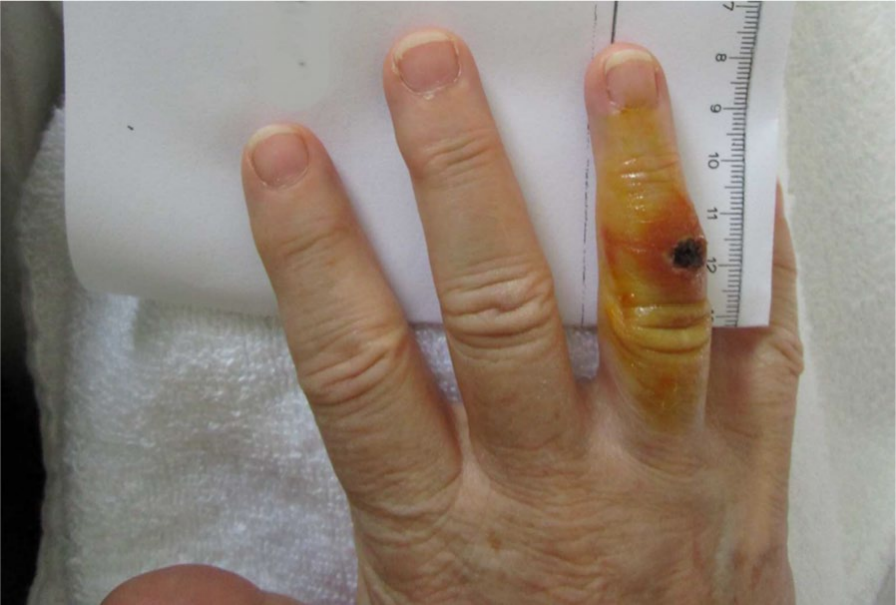
Picture of the right hand of the patient at day 14 after the squirrel bite on the digit IV. After presentation of the ulcerous lesion and the local lymphadenitis of the right elbow in combination with the non-effective antibiotic treatment using three different antibiotics over 2 weeks, the suspicion of an ulcero-glandular tularemia was expressed

In parallel, the wound on the patient’s right hand developed an ulcer-like lesion showing swollen reddened edges and a central incrustation (Fig. 1). The lymphadenitis of the right elbow persisted. It was at this stage that tularemia was suspected as a possible diagnosis for the first time. Following a phone call with the reference laboratory for human tularemia at the Robert Koch Institute (RKI) on day 14, the decision was made to send a wound swab sample from digit IV of the patient’s right hand (A-1825/1) to the Robert Koch Institute (RKI) for microbiological examination. In addition, a serum sample (A-1825/2) was taken for antibody detection (day 14). An enzyme-linked immunosorbent assay (ELISA) showed that antibodies against the lipopolysaccharide of *F. tularensis* Igpoly, immunoglobulin (Ig)G and IgM were borderline.

To isolate living bacteria and extract DNA, the wound swab was suspended in 900 µL double-distilled water, and a 450-µL of the suspension was centrifuged. The resulting pellet was used for genomic desoxyribonucleic acid (DNA) extraction (DNeasy Blood and Tissue kit; Qiagen, Hilden, Germany). A real-time PCR based on *F. tularensis*-specific primers (for genes *fopA* and *tul4*) confirmed the presence of *F. tularensis* DNA in the wound swab sample. Subspecies *F*th was identified by block PCR targeting the region of difference 1 (RD1) according to protocols described by Broekhuijsen *et al.* [15]. For culture, 50 µL of the swab suspension was inoculated in 10 mL medium T [16] and streaked onto MTKH [17], as well as onto chocolate agar plates and Neisseria selective medium plus (both Oxoid Germany GmbH, Wesel, Germany). The liquid medium and the agar plates were incubated at 37 °C with 5% CO_2_. Bacterial growth typical for *F. tularensis* could be observed on MTKH on day 3 of culture and on the chocolate and Neisseria agar plates on day 4. The patient’s cerebrospinal fluid did not test positive for *F. tularensis*.

**Fig. 2:**
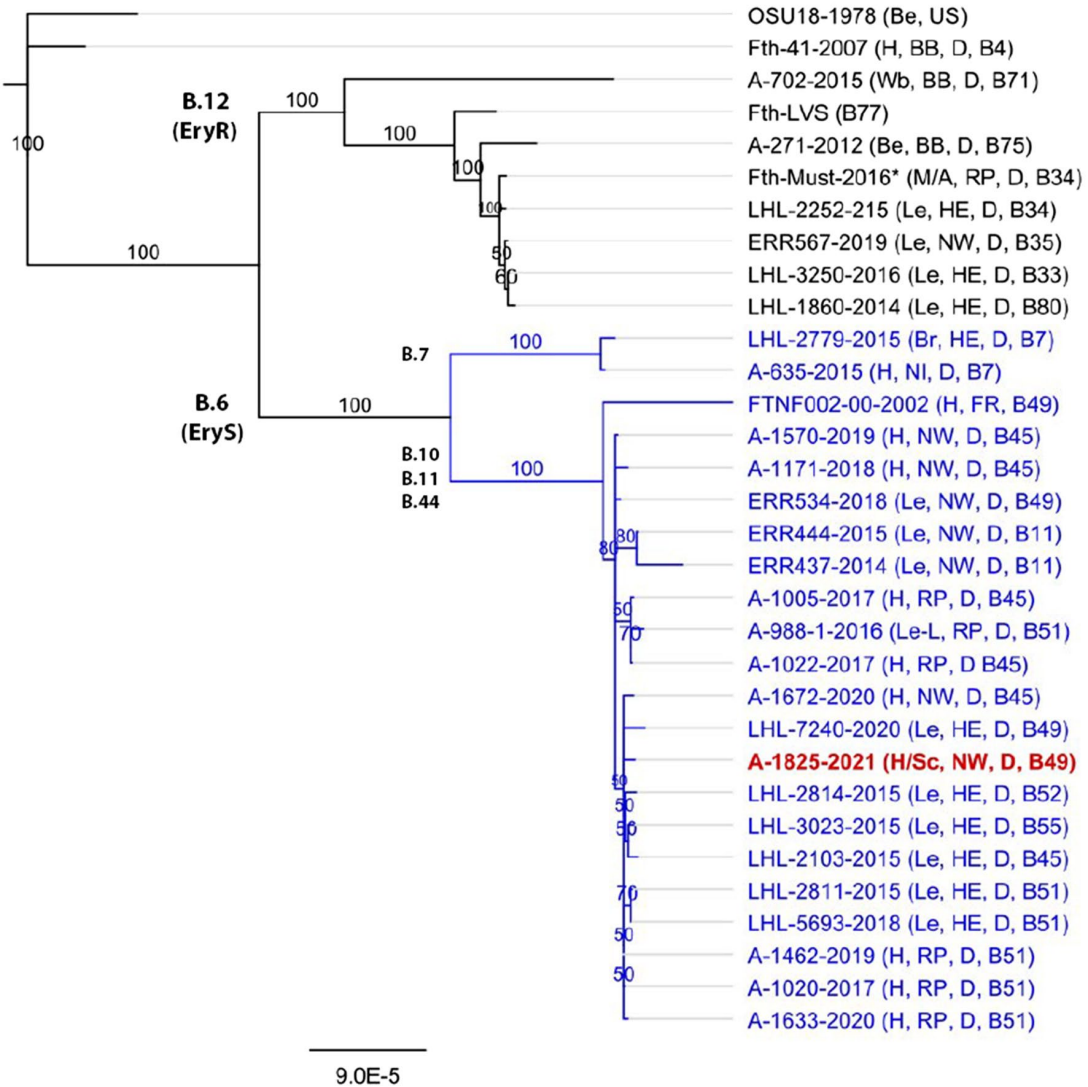
Phylogenetic relationship of *Francisella tularensis* ssp. *holarctica* (*Fth*) in a wound swab sample from digit IV of the patient’s right hand (A-1825/1) (red, in bold) to *F*th isolates from North Rhine-Westphalia (NW), Rhineland-Palatinate (RP), and Hesse (He). Strains belonging to the erythromycin-sensitive major clade B.6 are indicated in blue. For each genome sequence, the year of sampling, the host organism, sampling spot (German federal states), and the known final subclade are given. Host organisms: *Be* beaver, *Br* brock, *H* human, *Le* Lepus, *Sc* Sciurus, *Wb* wild boar. Germany’s federal states: *BB* Brandenburg, *HE* Hesse, *NI* Lower Saxony, *NW* North Rhine-Westphalia, *RP* Rhineland-Palatinate. Countries: *D* Germany, *FR* France. Asterisk indicates no bacterial isolate, genomic DNA only; for further details, see [13]. The analysis was based on a Mauve alignment for colinear genomes. Genomes were generated by DNA sequencing and mapping of DNA reads to the genome of *F. tularensis* ssp. *holarctica* (*F*th) strain LVS (for details, see [19]). For the clustering, the neighbor-joining bootstrap method was used, with *F. tularensis* ssp. *holarctica* (*F*th) strain OSU18 as an outgroup

Minimal inhibitory concentration testing according to the Clinical and Laboratory Standards Institute (CLSI; https://clsi.org/standards/products/microbiology/documents/m100/) guidelines for *F. tularensis* demonstrated an *in vitro* sensitivity to all antibiotics recommended for treatment of tularemia (for example, ciprofloxacin, gentamycin, levofloxacin) [6, 18]. The patient’s antibiotic treatment applied until that stage turned out to be inadequate (resistance against ampicillin/sulbactam, and cefuroxime, piperacillin/tazobactam are ineffective in the treatment for tularemia). In a sequencing study (MiSeq sequencer [Illumina Inc., San Diego, CA, USA], see [13] for details) and the phylogenetic analysis (Geneious prime [Biomatters, Auckland, New Zealand], Mauve alignment, followed by neighbor-joining clustering, see [19] for details), the DNA from *Fth* strain A-1825/1, isolated from the patient’s wound swab, was shown to belong to biovar I, clade B.6 and subclade B.49 (Fig. 2).

When the reference laboratory for human tularemia underpinned the suspected diagnosis on day 14, the treatment was immediately adjusted, and 500 mg of ciprofloxacin was administered twice a day for 14 days. Under the adjusted treatment, the patient recovered immediately (the CRP dropped to 15 mg/L) and she could be discharged fever-free with a negligible headache on day 17.

## Discussion and conclusions

We report here the case of an ulcero-glandular tularemia after a squirrel bite*.* Healthcare staff treat persons with animal bites, mainly by dogs, cats, and snakes [20], on a daily basis. In such cases, the risk of a systemic infection depends on a number of different parameters, such as the type of injury, the animal involved, and the patient’s own immune defense. The treatment should include immediate irrigation and debridement of the wound, a prophylaxis against tetanus, and possibly against rabies, and an antibiotic treatment (depending on the risk for a systemic infection) [21]. Although the risk of being bitten and/or infected by a wild Eurasian red squirrel (*Sciurus vulgaris*) is low [22], Europe has seen an increase in the number of tularemia cases in both humans and animals during the past decades [2, 7, 8]. While only one report of a natural infection with *Fth* in a wild Eurasian red squirrel, in Switzerland, has been published [14], it is known that squirrels, although being herbivores, may be a reservoir of *F. tularensis*, as are many other rodents. The human case of tularemia reported here from Germany also confirms the possibility of a European squirrel infected by *Fth*. After contact with squirrels, tularemia should, therefore, be considered as a differential diagnosis to prevent a delay of specific treatment [9, 10, 13].

Studies on *Francisella* isolates from humans and wild animals in Germany have revealed an unexpected genetic diversity of *F*th [19, 23–25] that is not only of academic interest: the phylogenetic analysis showed that *Fth* isolates of biovar I are erythromycin-susceptible and mainly occur in western Europe, whereas isolates of biovar II are erythromycin-resistant and mainly occur in northern and eastern Europe. A similar north-west divide has been observed in Germany [7, 19, 26–32]. The phylogenetic analysis of the draft genome sequence of strain A-1825 reported here, isolated from the patient in the Rhein-Erft region (Rhein-Erft-Kreis) in North Rhine-Westphalia, revealed that it belongs to erythromycin-sensitive biovar I (major clade B.6), subclade B.49 (Fig. 2). As mentioned, this is not surprising as *F*th isolates of biovar I appear more often in the south-west of Germany. The same applies to B.6 strains being more often detected in Rhineland-Palatinate and Hesse, near the Rhein-Erft region [7, 19, 28].

The clinical manifestation of tularemia depends on the portal of entry of the bacteria into the organism. Consequently, the disease is defined by the following different forms: ulcero-glandular or glandular, oropharyngeal, ocular-glandular, and respiratory [1, 3, 6]. The ulcero-glandular form of tularemia mostly occurs after direct skin contact with an infected animal or by percutaneous penetration of the pathogen after a bite by a wild or domestic animal (cats, dogs) or by a tick [2, 33–37]. The following primary clinical symptoms may occur 1–14 days (mainly 3–5 days) after penetration of the pathogen: fever, malaise, headache, melalgia, and swollen lymph nodes [6, 8, 18]). Subsequently, an ulcerous lesion at the portal of entry and swollen lymph nodes may appear. The ocular-glandular form can be caused by eye contact with contaminated materials and may result in a conjunctivitis. The oropharyngeal form is characterized by a mostly unilateral cervical lymph node swelling, stomatitis, tonsillitis, and pharyngitis after ingestion of contaminated food or water. The respiratory form of tularemia after inhalation of the pathogen may cause cough, chest pain, dyspnea, and pneumonia [6]. For diagnosis, serological methods, antigen detection, or molecular methods are used [38]. Specific real-time PCR assays and bacterial growth on suitable agar plates (for example, chocolate, Neisseria selective medium plus) allow the pathogen to be identified in clinical material, such as wound swabs or blood cultures [38]. If an isolate is available, mass-assisted laser desorption/ionization time-of-flight spectrometry can be used for identification of *F. tularensis*. Whole-genome sequencing and PCR targeting the RD1 for subspecies differentiation [15] are methods which enable further characterization of the pathogen [3]. Serological methods, for example ELISA or western blot, can be used for antibody detection against the lipopolysaccharide of *F. tularensis* to confirm tularemia retrospectively, but only if the incident occurred more than 2–3 weeks prior to the tests or assays.

The clinical presentation of this case in combination with the ineffective antibiotic treatment, namely, three different antibiotics in 2 weeks, underpinned the suspected diagnosis of an ulcero-glandular form of tularemia proposed by clinicians and specialists from the reference laboratory. After the appropriate antibiotic treatment with ciprofloxacin, the patient recovered immediately and could be discharged from hospital on day 3 after treatment adaptation (aminoglycosides, quinolones, tetracyclines, and chloramphenicol) [37].

## Data Availability

Not applicable.
